# Women’s Perceptions of Medication Use During Pregnancy and Breastfeeding in Saudi Arabia

**DOI:** 10.7759/cureus.32953

**Published:** 2022-12-26

**Authors:** Namshah A Alhajri, Aljohara H Alshathri, Sarah S Aldharman, Almaha H Alshathri, Jana K Abukhlaled, Durrah W Alabdullah, Sarah A Aleban

**Affiliations:** 1 Department of Obstetric and Gynecology, Princess Nourah Bint Abdulrahman University, Riyadh, SAU; 2 College of Medicine, King Saud University, Riyadh, SAU; 3 College of Medicine, King Saud bin Abdulaziz University for Health Sciences, Riyadh, SAU; 4 College of Medicine, Princess Nourah Bint Abdulrahman University, Riyadh, SAU

**Keywords:** saudi arabia, breastfeeding, pregnancy, medication, perceptions, women's

## Abstract

Background

Pregnancy is a unique physiological condition in which medication intake offers a challenge and a worry due to changed drug pharmacokinetics and drugs potentially crossing the placenta, such as beta blockers and benzodiazepines. As a result, medication safety during pregnancy has gained global interest, attracting attention from doctors and pregnant women, little of which has been documented regarding the Saudi population. Therefore, this study aimed to assess medication use and perceptions of medication use during pregnancy and breastfeeding among women in Saudi Arabia.

Methods

This study is a questionnaire-based, cross-sectional study. Data was collected through an online self-administered questionnaire from different regions of Saudi Arabia. Data was then entered and analyzed using SPSS 24.0 version (IBM Inc., Chicago, USA) statistical software.

Results

A total of 1831 participants were included in the current study. About 835 (45.6%) of the participants were within the age group of 26-35 years old. A total of 602 (32.9%) were using medications on a daily basis or several times a week during pregnancy or breastfeeding. About 1476 (80.6%) participants agreed on medication use during pregnancy. About 66.4% of women would be worried about fetal malformations if they were supposed to take medications during pregnancy. About 940 (51.3%) women think that medication use during early pregnancy is harmful, and 500 (27.3%) think that medication use during breastfeeding trimesters is harmful. Regarding pregnant women's perception of herbal medicines, about (65.4%) of those with low educational levels think that herbal medicines are harmful in early pregnancy. Most participants (63%) within the age group of ≥36 years old think that medications and herbal medicines are harmful in early pregnancy. The vast majority (91%) of the participants would ask the physician working on antenatal care if they had concerns about using certain medications during pregnancy.

Conclusion

The average use of medication and herbal medicines among pregnant women was noted, although many women had negative beliefs about taking certain medications. Continued effort is essential to support and encourage women to seek out reliable information sources regarding medication use during pregnancy. In addition, healthcare practitioners should be mindful of women's attitudes when counseling them to take medication during pregnancy.

## Introduction

Pregnant women experience various symptoms during pregnancy [1]. Some medications are used during pregnancy to treat various symptoms and diseases [1]. Some women with previously diagnosed medical conditions such as asthma, diabetes mellitus, or any autoimmune disease such as systemic lupus should continue the prescribed medications to avoid adverse pregnancy outcomes, including stillbirth, low birth weight, or preeclampsia [2]. Likewise, adherence to the appropriate treatment regimen throughout pregnancy is critical for pregnant women with HIV to reduce the risk of viral transmission to the fetus [3]. Perception of teratogenic risk and maternal decision-making may be influenced by multiple factors, such as maternal emotional state, opinions, experiences, and beliefs of their family and friends [4,5]. There is little research on pregnant women's perceptions of medicine use. However, to our knowledge, these studies only provide scant information about the pregnant population in Saudi Arabia [6-8]. Other studies have indicated that the parity and educational status of pregnant women can influence how pregnant women view the use of medications, but the outcomes are diverse [6,9,10]. For example, a study conducted among Norwegian women showed that higher-educated women were more hesitant to take any medication during pregnancy. In contrast, less-educated women believed that pharmaceuticals, in general, were hazardous and herbal medicines were safe [9]. However, another study conducted in Sweden showed that education level or parity did not affect attitudes toward medicine use generally during pregnancy and breastfeeding [1]. It also showed that most pregnant women believed that taking medications during pregnancy or while breastfeeding was probably harmful or harmful [1].

Another important health issue is the rise in the use of herbal medicines. Despite possible maternofetal dangers, many pregnant women still utilize herbal remedies. For example, there was a study conducted in Hail in Saudi Arabia about the use of herbal medicine during pregnancy. The findings of this study indicated a high prevalence of herbal medicine use during pregnancy in the region, most likely influenced by local cultural practices. The majority of participants believed that moderate use of herbal medications would not cause any harm to the mother or the fetus. However, they believed excessive use would result in unfavorable outcomes such as stillbirth, early labor, infant abnormalities, and long-term repercussions on maternal health [11]. Using medication during pregnancy is a concern for most pregnant women, either over the counter or prescribed medications [4]. Understanding these concerns may lead to achieving the best counseling for pregnant women, resulting in better adherence to their medications and preventing negative outcomes [1]. Therefore, this study aimed to investigate women's perceptions of medication use during pregnancy and breastfeeding in Saudi Arabia.

## Materials and methods

This descriptive cross-sectional questionnaire-based study was conducted in Saudi Arabia between August 2022 and November 2022. The target subjects were females from the general population of Saudi Arabia from different regions (central, Southern, Eastern, Western, and Northern). The study was conducted through an online self-administered questionnaire. The questionnaire was distributed on different online platforms, so the data was collected from different regions of Saudi Arabia. All responses were collected and exported into a Microsoft Excel spreadsheet file by Google Docs tools for processing and analyzing information. Data were analyzed using SPSS 24.0 version (IBM Inc., Chicago, USA) statistical software.

Sample size and sampling technique

OpenEpi® version 3.0 software was employed to calculate the sample size, which is representative of Saudi Arabia's female population of 15 million. The representative sample size required was 385, with a margin error determined as 5% and a confidence level determined as 95%. We aimed to obtain more than the calculated sample size to overcome any exclusions. Therefore, non-probability convenience sampling techniques have been employed.

Inclusion criteria and exclusion criteria

The study's eligibility criteria included Saudi females aged 18 years and above who are pregnant or have been pregnant before. Participants who did not complete the questionnaire or did not agree to participate were excluded. 

Data collection instrument and procedures

A validated self-questionnaire from a previous study was used [1]. In addition, a cover letter with instructions and information about medication definitions (i.e., not to include iron and vitamin supplements as medication; herbal medicines were defined as products for self-care with animal or plant origins and sold over the counter) was used. The questionnaire contained questions regarding demographics including age, parity, educational levels, and current medication use: frequent users (medication use daily to several times a week), non-frequent users (medication use once a week to once a month), and non-users (medication use rarely or never). Also, the questionnaire contained questions asking about pregnant women's perceptions of medication use and herbal medicines in general and their perceptions about medication use in relation to the need for the medication. Lastly, the questionnaire had a question asking about the sources of information related to medications. 

The questionnaire was translated into Arabic by an expert to make it easier for the target population to read and understand. In addition, an electronic Google Forms survey was used and distributed on different social media platforms such as WhatsApp, Twitter, and Telegram. Features of Google Forms, such as "required to proceed" to ensure the study criteria would be fulfilled, were employed. The following question was provided at the beginning of the questionnaire: "Are you pregnant now, or have you ever been pregnant?" If the answer were "yes," the participant would continue to go through questions in the questionnaire; however, if the answer were "no," the questionnaire form would be submitted directly.

All information was kept private and used for scientific research. Participation in this study was voluntary and optional, with informed consent provided to all participants on the first page before filling out the questionnaire. The ethical approval of the study was obtained before initiating the study. The ethical approval was obtained from Institutional Review Board (IRB) at Princess Nourah Bint Abdulrahman University (reference No. 22-0393).

Statistical analysis

The collected data was first entered into a Microsoft Excel file and later transferred to SPSS 24.0 version (IBM Inc., Chicago, USA) statistical software for further analysis. The mean ± SD was reported for continuous variables, while categorical variables were described using frequencies and percentages. A Chi-square test was used to compare categorical variables. A p-value <0.05 was considered significant.

## Results

Characteristics of the participants

A total of 1831 participants were enrolled in the current study. In regard to age groups, 835 (45.6%) of the participants were within the age group of 26-35 years old, 539 (29.4%) were within the age group of 36-45 years old, 263 (14.4%) were within the age group of 18-25 years old and 194 (10.6%) were more than 45 years old. The educational level of most of the participants was as follows, 1288 (70.3%) had a bachelor's degree, 405 (22.1%) had a public education, and 138 (7.5%) had a postgraduate educational level. A total of 1328 (72.5%) were multipara, and 503 (27.5%) were primipara. In addition, 602 (32.9%) were using medications on a daily basis or several times a week, 401 (21.9%) were found to be using medications once a week or once per month, whereas 828 (45.2%) rarely or never used medications during pregnancy or breastfeeding. The characteristics of the study participants are shown in Table 1.

**Table 1:** Characteristics of the study participants (n = 1831).

Variable	Category	Frequency	Percent
Age (years)	18-25	263	14.4%
	26-35	835	45.6%
	36-45	539	29.4%
	> 45	194	10.6%
Educational Level	Public education	405	22.1%
	Bachelor’s degree	1288	70.3%
	Postgraduate degree	138	7.5%
Parity	Primipara	503	27.5%
	Multipara	1328	72.5%
Use of medication	Daily - several times a week	602	32.9%
	Once a week - once a month	401	21.9%
	More rarely - never	828	45.2%

About 1476 (80.6%) of the participants agreed on medication use during pregnancy, 193 (10.5%) disagreed on the use of medications during pregnancy, whereas 162 (8.8%) were with no opinion. A total of 803 (43.9%) of the participants agreed that women should avoid all kinds of medications during pregnancy. About 336 (18.4%) participants agreed that they tend to use medications more during pregnancy. About 802 (43.8%) of the participants agreed that using medications during pregnancy does more harm than good. A total of 1572 (85.9%) would stick to treatment if prescribed medications during pregnancy, whereas 175 (9.6%) would not stick to prescribed medications during pregnancy, and 84 (4.6%) were with no opinion. About 922 (50.4%) of the participants agreed that women's health is prioritized when medications are used during pregnancy over the health of the fetus, 555 (30.3%) did not agree with the same statement, whereas 354 (19.3%) were with no opinion. Table 2 illustrates pregnant women's perceptions of medication use in general.

**Table 2:** Pregnant women's perceptions about medication use in general.

Statement	Agree/Partly agree	Disagree/Partly disagree	No opinion
	N (%)		
My view of medication use during pregnancy has changed since I became pregnant.	1476 (80.6)	193 (10.5)	162 (8.8)
Pregnant women should avoid all kinds of medication	803 (43.9)	843 (46)	185 (10.1)
I tend to use medication when I'm pregnant more than I tend to use medication when I'm not	336 (18.4)	1356 (74.1)	139 (7.6)
Using medication during pregnancy does more harm than good	802 (43.8)	637 (34.8)	392 (21.4)
If prescribed medication during pregnancy, I will stick to the treatment	1572 (85.9)	175 (9.6)	84 (4.6)
Women's health is prioritized when medication is used during pregnancy over the health of the fetus	922 (50.4)	555 (30.3)	354 (19.3)

**Figure 1:**
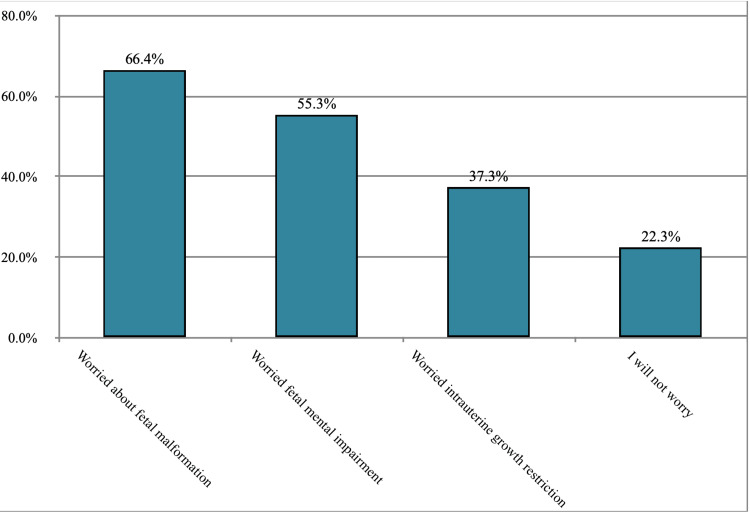
The worries of the pregnant women while taking medication during pregnancy.

About 66.4% of women would be worried about fetal malformations if they were supposed to take medications during pregnancy, 55.3% of the participants would be worried about fetal mental impairment, 37.3% would be worried about intrauterine growth restriction, and 22.3% would not be worried at all. Figure 1 shows the pregnant woman's worries while taking medication during pregnancy.

About 940 (51.3%) women think that medication use during early pregnancy is harmful, 744 (40.6%) think that medications during early pregnancy are probably harmful, 116 (6.3%) think that it is probably harmless, and 31 (1.7%) think that medications during early pregnancy are harmless. Frequent medication use was found to be linked with perceptions about medication use during pregnancy (p-value= 0.019), with frequent medication users thinking that medications are not harmful compared to others. About 441 (24.1%) women think that medication use during the second and third trimesters is harmful, 874 (47.7%) think that medications during early pregnancy are probably harmful, 444 (24.2%) think that it is probably harmless, and 72 (3.9%) think that medications during second and third trimesters of pregnancy are harmless. Frequent medication use was found to be linked with perceptions about medication use during pregnancy (p-value= 0.004), with frequent medication users thinking that medications are not harmful compared to others. A total of 500 (27.3%) women think that medication use during breastfeeding trimesters is harmful, 924 (50.5%) think that medications during breastfeeding are probably harmful, 444 (24.2%) think that it is probably harmless, and 87 (4.8%) think that medications during breastfeeding are harmless.

About 1077 (58.8%) women think that herbal medicine use during early pregnancy is harmful, 596 (32.6%) think that herbal medicine use during early pregnancy is probably harmful, 120 (6.6%) think that it is probably harmless, and 38 (2.1%) think that herbal medicine during breastfeeding is harmless. A total of 707 (38.6%) women think that herbal medicine use during late pregnancy is harmful, 759 (41.5%) think that herbal medicine use in late pregnancy is probably harmful, 304 (16.6%) think that it is probably harmless, and 61 (3.3%) think that herbal medicine during late pregnancy is harmless. About 468 (25.6%) women think that herbal medicine use during breastfeeding is harmful, 766 (41.8%) think that herbal medicine use during breastfeeding is probably harmful, 428 (23.4%) think that it is probably harmless, and 169 (9.2%) think that herbal medicine during breastfeeding is harmless. The pregnant women's perceptions about medication use and herbal medicines in general in relation to their own medication use are shown in Table 3.

**Table 3:** Pregnant women's perceptions about medication use and herbal medicines in general in relation to their own medication use.

Statement	Harmful n (%)	Probably harmful n (%)	Probably harmless n (%)	Harmless n (%)	P-value
What are your perceptions about medication use during early pregnancy (1^st^ trimester)?					
All women	940 (51.3)	744 (40.6)	116 (6.3)	31 (1.7)	
Frequent medication users	290 (48.2)	246 (40.9)	51 (8.5)	15 (2.5)	0.019
Non-frequent medication users	198 (49.4)	171 (42.6)	26 (6.5)	6 (1.5)	
Non-users	452 (54.6)	327 (39.5)	39 (4.7)	10 (1.2)	
What are your perceptions about medication use during late pregnancy (2^nd^ & 3^rd^ trimesters)?					
All women	441 (24.1)	874 (47.7)	444 (24.2)	72 (3.9)	
Frequent medication users	138 (22.9)	262 (43.5)	169 (28.1)	33 (5.5)	0.004
Non-frequent medication users	88 (21.9)	198 (49.4)	98 (24.4)	17 (4.2)	
Non-users	215 (26)	414 (50)	177 (21.4)	22 (2.7)	
What are your perceptions about medication use during breastfeeding and the effect on the child?					
All women	500 (27.3)	924 (50.5)	320 (17.5)	87 (4.8)	
Frequent medication users	164 (27.2)	280 (46.5)	119 (19.8)	39 (6.5)	0.049
Non-frequent medication users	118 (29.4)	206 (51.4)	61 (15.2)	16 (4)	
Non-users	218 (29.3)	438 (52.9)	140 (16.9)	32 (3.9)	
What are your perceptions about herbal medicine use during early pregnancy (1^st^ trimester)?					
All women	1077 (58.8)	596 (32.6)	120 (6.6)	38 (2.1)	
Frequent medication users	361 (60)	187 (31.1)	48 (8)	6 (1)	0.044
Non-frequent medication users	227 (56.6)	133 (33.2)	26 (6.5)	15 (3.7)	
Non-users	489 (59.1)	276 (33.3)	46 (5.6)	17 (2.1)	
What are your perceptions about herbal medicine use during late pregnancy (2^nd^ & 3^rd^ trimesters)?					
All women	707 (38.6)	759 (41.5)	304 (16.6)	61 (3.3)	
Frequent medication users	223 (37)	249 (41.4)	110 (18.3)	20 (3.3)	0.719
Non-frequent medication users	154 (38.4)	167 (41.6)	63 (15.7)	17 (4.2)	
Non-users	330 (39.9)	343 (41.4)	131 (15.8)	24 (2.9)	
What are your perceptions about herbal medicine use during breastfeeding and the effect on the child?					
All women	468 (25.6)	766 (41.8)	428 (23.4)	169 (9.2)	
Frequent medication users	154 (25.6)	240 (39.9)	152 (25.2)	56 (9.3)	0.044
Non-frequent medication users	101 (25.2)	155 (38.7)	94 (23.4)	51 (12.7)	
Non-users	213 (25.7)	371 (44.8)	182 (22)	62 (7.5)	

About 306 (73.9%) of the participants with asthma or allergy needed the medications and used them, but 108 (26.1%) refrained from using them. A total of 275 (17.1%) participants needed to use paracetamol during pregnancy but refrained from using it, and 1332 (82.9%) used it. A total of 263 (25.1%) needed medications for heartburn and gastritis but refrained from using them, whereas 784 (74.9%) needed the medications and used them. About 257 (79.1%) of the participants will use medications necessary for an illness and strongly recommended. About 255 (71%) needed medications for chronic pain and used them during pregnancy, and 104 (29%) refrained from using them. Depression medications were used by 75 (46.3%) participants who needed them during pregnancy, whereas 87 (53.7%) needed the medication but refrained from using it. Medications for headaches or migraine were needed and used by 505 (66.9%) participants, and 250 (33.1%) needed the medications but refrained from use. About 709 (72.9%) of the participants needed antibiotics and used them during pregnancy, but 264 (27.1%) participants needed antibiotics but refrained from using them. Table 4 shows the pregnant women's perceptions about medication use in relation to the need for the medication.

**Table 4:** Pregnant women's perceptions about medication use in relation to the need for the medication.

Do you need to use the following medications while pregnant or breastfeeding?	Have needed the medication but refrained from use	Have needed the medication and used it
	n (%)	
Asthma/Allergy	108 (26.1)	306 (73.9)
Paracetamol	275 (17.1)	1332 (82.9)
Heartburn/Gastritis	263 (25.1)	784 (74.9)
An illness where medication treatment is necessary or strongly recommended	68 (20.9)	257 (79.1)
Chronic pain	104 (29)	255 (71)
Depression	87 (53.7)	75 (46.3)
Headache or migraine	250 (33.1)	505 (66.9)
Antibiotics	264 (27.1)	709 (72.9)

**Figure 2:**
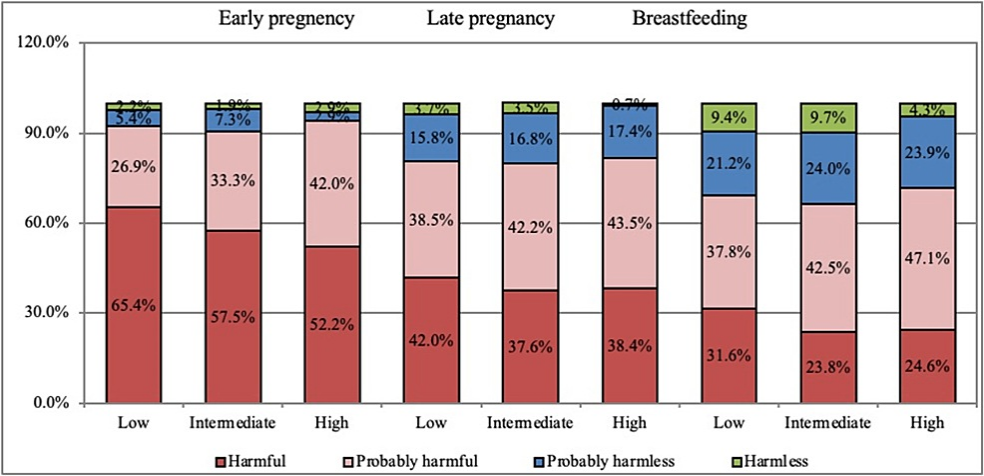
Pregnant women's perceptions about herbal medicines in relation to the level of education. Educational levels are divided into three groups (Low = Public education, Intermediate = Bachelor’s degree, High = Postgraduate degree).

In regard to pregnant women's perception of herbal medicines, about 65.4% of those with a low educational level think that herbal medicines are harmful in early pregnancy, 57.5% of those with an intermediate educational level think that it is harmful, and 52.2% of those with high educational level in early pregnancy think that it is harmful (p-value = 0.007). About 42.0% of those with a low educational level think that herbal medicines are harmful in late pregnancy, 37.6% of those with an intermediate educational level think that it is harmful, and 38.4% of those with a high educational level think that herbal medicines are harmful in late pregnancy (p-value = 0.424). A percentage of 31.6% of those with a low educational level think that herbal medicines are harmful in breastfeeding, 23.8% of those with an intermediate educational level think that it is harmful in breastfeeding, and 24.6% of those with a high educational level think that herbal medicines are harmful in breastfeeding (p-value = 0.020). Figure 2 illustrates pregnant women's perceptions of herbal medicines in relation to their level of education.

**Figure 3:**
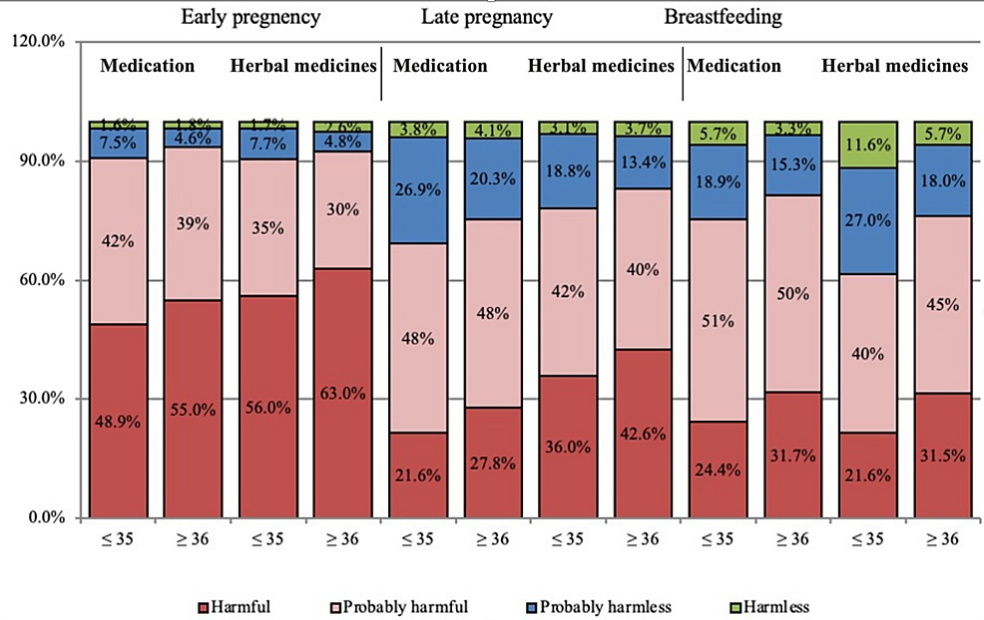
Pregnant women's perceptions about medication and herbal medicines in relation to age.

Most of the participants (63%) within the age group of ≥36 years old think that herbal medicines are harmful in early pregnancy (p-value = 0.002). Also, most of them (55%) think that medications are harmful in early pregnancy (p-value = 0.019) compared to participants within the age group of ≤35. Also, most of the participants (42.6%) within the age group of ≥36 years old thought that herbal medicines are harmful in late pregnancy (p-value = 0.004), and a higher percentage of them (27.8%) within the age group of ≥36 years old thought that medications are harmful in late pregnancy (p-value = 0.002). In addition, 31.5% of the participants within the age group of ≥36 years old think that herbal medicines are harmful during breastfeeding (p-value < 0.001). Also, a higher percentage of them (31.7%) think that medications are harmful during breastfeeding (p-value = 0.001) compared to participants within the age group of ≤35. Figure 3 presents pregnant women's perceptions of medication and herbal medicines in relation to age.

**Figure 4:**
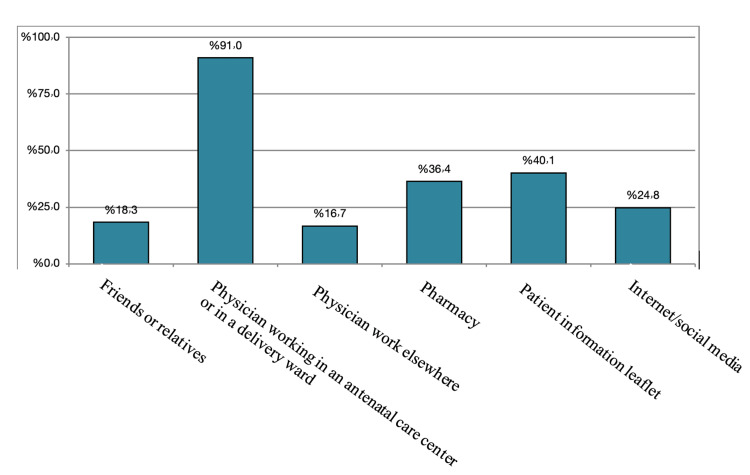
The source of information regarding medication use during pregnancy.

The vast majority (91%) of the participants would ask the physician working on antenatal care if they had concerns about the use of certain medications during pregnancy. A total of 40.1% will turn to patient information leaflets, 36.4% will go to the pharmacy, 24.8% will search on the internet or social media, 18.3% will ask friends or relatives, and 16.7% will ask a physician work elsewhere. Figure 4 illustrates the source of information regarding medication use during pregnancy.

## Discussion

Assessing perceptions about medication use and risks among women during pregnancy is essential, as many pregnant women may have various medical issues which need to be addressed and treated with certain medications. Women's perception of medication significantly affects their choices and subsequently influence their life with certain medical conditions [12]. This study aimed to assess the perceptions of medication use during pregnancy and breastfeeding among women in Saudi Arabia.

Nearly 32.9% of the participants in this study used medication daily or several times a week. About 21.9% used medications once a week or once per month, whereas 45.2% rarely or never used medications during pregnancy or breastfeeding. This conclusion is similar to another research conducted in Sweden, where 50% of pregnant women filled a prescription at least once [1]. This demonstrates the importance of researching pregnant women's attitudes toward medication use to provide them with expert counseling.

Previous research has shown that women become more cautious about using medications while pregnant [6,9]. However, most of the women in our research had changed their perceptions about using medications since getting pregnant, and most of them felt that pregnant women should abstain from taking any sort of medicine. Additionally, they were less likely to take medicine when pregnant than when not. This suggests that detailed medication counseling is required during pregnancy, especially when a woman is given a prescription for a medication that is critical for her health or the health of the unborn child. In our study, about (43.8%) of pregnant women agreed that taking medications during pregnancy or while breastfeeding was dangerous and did more harm than good. About 47.7% of our participants think using medications during early pregnancy is probably harmful. This was supported by another study done in Sweden, where more than 50% of pregnant women thought that taking medications in the first trimester, last trimester, or while breastfeeding was dangerous or probably detrimental [1]. We found that those with low educational levels think that herbal medicines are harmful in early pregnancy. For example, a study done in Saudi Arabia stated that pregnant women with low levels of education were more comfortable using herbal and/or natural medicines during their pregnancies and believed they did not need to visit a doctor first [6]. Similarly, prior research has shown that women with a lower level of education regarded using herbal medicines as more dangerous than women with a higher level of education [9].

We found that nearly two-thirds (66.4%) of the women admitted that if they took medication while pregnant, they would be concerned about fetal malformation. This shows the need to provide pregnant women with accurate and balanced information about the potential effects of medications on the fetus's health. As a result, inquiries regarding potential worry should be carefully addressed and answered. These findings are consistent with the Swedish study [1]. Also, this was consistent with the findings reported in the congruent study conducted in the Netherlands, in which the most reported maternal concerns had a child with congenital disabilities [13]. An online survey of 210 pregnant women revealed that 36.2% had low medication adherence. Low adherence to the treatment plan during pregnancy was largely influenced by preconceptions about medications [14]. Similarly, a small number of participants in our study declined to receive necessary or highly recommended medicine. The pregnant women in our study appeared to prefer consulting with physicians who worked in antenatal care facilities for guidance and information regarding medication use. Given that the women in this study showed a high level of confidence in the advice of healthcare experts, asking directly about worries regarding medication use can likely result in better adherence to a treatment regimen. In our study, most women chose professional information sources (physicians) above unofficial sources (i.e., the internet and friends/relatives). Another study done in Sweden had the same results as our study [1]. Also, this was consistent with the findings of the Ethiopian study, which also concluded that physician was the most reported source of information [15]. 

Age and attitudes toward using herbal medicines were also found to be significantly correlated; younger women evaluated the use of herbal medicines during pregnancy as less dangerous than older women. Even though little is known about herbal remedies' potential side effects, teratogenicity, and drug interactions [6], the pregnant women in our study considered herbal medicines less harmful than medications.

A previous study on the Saudi population showed that most women generally believed that medications are not harmful. This is inconsistent with our study, which showed that about 940 (51.3%) women think that medications used during early pregnancy are harmful [6]. In comparison, another study conducted in Al-Ahsa in Saudi Arabia [7] showed that more than half of the participants would stick to treatment if prescribed medications during pregnancy. This finding agrees with our results, in which the vast majority (85.9%) will stick to treatment if prescribed medications during pregnancy. In addition, most of our participants, 1332 (82.9%), used paracetamol, and according to the FDA's classification of drug risks, paracetamol is in category B, which is generally regarded as safe during pregnancy [7].

The majority (80.6%) of the participants agreed on medication use during pregnancy. Less than half (43.8%) of the participants agreed that using medications during pregnancy does more harm than good. Half (50.4%) of the participants agreed that women's health is prioritized when medications are used during pregnancy over the health of the fetus. These findings were found to be similar to the findings from the congruent study carried out in the Italian study, which also reported that more than half of pregnant women agreed to use medications during pregnancy for their health [16].

More than two-thirds (79.1%) of the participants will use medications if necessary for an illness and strongly recommended by doctors. Similar findings were reported in the parallel study carried out in Ethiopia, where most participants do not take medications during pregnancy without medical advice [15].

Given the inclusion criteria and the high response rate, the likelihood of response bias is reduced, which makes the findings generalizable to a Saudi population. However, only Saudi women who could read and write Arabic were included in the study, which presents a limitation. As a result, we cannot say with certainty if the findings apply to pregnant women who cannot read or write Arabic.

This study examined only those who got pregnant. Future research should include all women, whether they are planning to be pregnant or newly married, to measure women's perception of medication in a broader range. In addition, the present study assessed the women's perceptions regarding medications and herbal medicines use in general. We did not address specific medication use; therefore, we cannot comment on whether it is appropriate or problematic for pregnant women to avoid certain drugs. Nevertheless, we recommend adopting national programs to increase awareness about medication and supplementation during pregnancy. Moreover, further studies use cohorts or case-control to examine the effects of medication on the mother and the child during pregnancy and breastfeeding.

## Conclusions

The average use of medications and herbal medicines among pregnant women was observed; however, many women had negative beliefs about taking certain medications. The reasons for the perception were variable, but it was mainly worrying about fetal malformation and maternal impairment. Most pregnant women would ask the physician about possible concerns about medication during pregnancy. There was a significant relationship between age and the women's perceptions of medication and herbal medicines. More effort is recommended to educate and encourage women to obtain information regarding medication use during pregnancy and breastfeeding from verified sources.
